# Variants in the host genome may inhibit tumour growth in devil facial tumours: evidence from genome-wide association

**DOI:** 10.1038/s41598-017-00439-7

**Published:** 2017-03-24

**Authors:** Belinda Wright, Cali E. Willet, Rodrigo Hamede, Menna Jones, Katherine Belov, Claire M. Wade

**Affiliations:** 1 School of Life and Environmental Sciences, Faculty of Veterinary Science, University of Sydney, Sydney, NSW Australia; 2 Sydney Informatics Core Research Facility, University of Sydney, Sydney, New South Wales Australia; 3 School of Biological Sciences, University of Tasmania, Hobart, Tasmania Australia

## Abstract

Devil facial tumour disease (DFTD) has decimated wild populations of Tasmanian devils (*Sarcophilus harrisii*) due to its ability to avoid immune detection and pass from host to host by biting. A small number of devils have been observed to spontaneously recover from the disease which is otherwise fatal. We have sequenced the genomes of these rare cases and compared them to the genomes of devils who succumbed to the disease. Genome-wide association, based on this limited sampling, highlighted two key genomic regions potentially associated with ability to survive DFTD. Following targeted genotyping in additional samples, both of these loci remain significantly different between cases and controls, with the PAX3 locus retaining significance at the 0.001 level, though genome-wide significance was not achieved. We propose that PAX3 may be involved in a regulatory pathway that influences the slowing of tumour growth and may allow more time for an immune response to be mounted in animals with regressed tumours. This provides an intriguing hypothesis for further research and could provide a novel route of treatment for this devastating disease.

## Introduction

The Tasmanian devil (*Sarcophilus harrisii*), a carnivorous marsupial native to Australia, is threatened with extinction in the wild due to devil facial tumour disease (DFTD). DFTD is a rare form of transmissible cancer that was first officially observed in 1996 in the north-east of Tasmania^1^. The tumour is transmitted as an allograft by biting during social interactions^2, 3^. As DFTD arose once in a north-eastern devil and then spread throughout the population, it is unusual in that the tumour genome has a different origin to that of its host. Since its emergence, DFTD has rapidly spread throughout all but the far-western populations of devils with infection rates of up to 95%^4^. This high infection rate is in part due to the ability of the tumour to evade immune detection by down-regulating the major histocompatibility complex (MHC)^5^. Tumours usually develop on the face and metastasise to distal organs, resulting in organ failure and death^6, 7^. Examination of the tumour transcriptome has indicated a Schwann cell origin – a cell of the peripheral nerve with a neural crest precursor^8^. There is now evidence of evolution in response to DFTD with allele frequency changes over time following disease-mediated sweeps in wild devil populations^9^.

In one wild north-western Tasmanian population, West Pencil Pine (WPP)^10^, it has been observed that some devils have spontaneously cleared the cancer. These individuals were confirmed to have DFTD when first sampled, yet tumours were recorded to have shrunk or disappeared on subsequent sampling^11^. There are no apparent differences in MHC profiles within this population relating to DFTD infection^12^. Some devils from this population have demonstrated serum antibodies against DFTD^11^. The underlying cause of this rare immune response remains unclear. Our hypothesis is that variants within the genomes of these particular devils may confer some resistance to DFTD progression or metastasis.

To investigate this we compared the genomes of seven devils that survived DFTD infection or displayed evidence of tumour regression to six young control devils that succumbed to the disease. Young control devils were used to account for potential effects of immunological senescence increasing susceptibility to infection. Through comparisons with the Tasmanian devil reference genome^13^, we discovered variable sites across the genomes of these samples. We next employed a genome-wide association to identify genomic regions that segregate different haplotypes between young, DFTD-infected devils and devils with regressed tumours from the WPP population. Three candidate loci were further targeted in additional controls from the same population. We present two key regions of the genome that may contribute to the observed tumour regression in these devils.

## Results/Discussion

Genome-wide association (GWAS) analysis revealed a large region spanning 1.6 megabases (Mb) on chromosome 3. The region contains 38 single nucleotide polymorphisms (SNPs) that fall within the top 100 loci from association after filtering on quality, seven of which map within introns of the *PAX3* gene (best p value = 0.00007; Table 1, also Table S1). A second associated region on chromosome 6 spans 830 kilobases (kb). This region contains 17 SNPs within the top 100 associated loci after filtering and eight of these map to intronic sequence of the *TLL1* gene (best p value = 0.00022; Table 1, also Table S1). No SNPs affecting coding sequence were found within the associated regions in the genomes of the sampled devils.

**Table 1:** Summary of loci segregating with DFTD regression identified by GWAS. Key candidate genes for DFTD regression are in bold.

Scaffold	Scaffold Size (Mb)	No. of SNPs (In top 100)	Best p-value	INDELs	Nearest Candidate Genes
Chr1_0002	1.08	97 (2)	0.00039	189	MKI67
Chr1_0216	3.85	203 (1)	0.00094	443	RBL1
Chr1_0248	0.97	102 (1)	0.00049	217	CHRNA4
Chr1_0256	4.38	410 (1)	0.00094	1047	DOK5
Chr1_0386	0.71	136 (1)	**0**.**00006**	173	**NBAS**, DDX1
Chr2_0233	4.02	222 (2)	0.00045	562	YWHAZ
Chr2_0238	0.81	274 (1)	0.00094	411	EBAG9
Chr2_0398	3.39	419 (4)	0.00045	945	UNC13B
Chr3_0006	0.94	14 (1)	0.00067	74	SEPT2
Chr3_0048	2.23	96 (1)	0.00131	328	CD96
Chr3_0369	3.06	146 (2)	0.00028	451	STK11IP, INHA, OBSL1
Chr3_0388	4.28	407 (38)	**0**.**00007**	1496	**PAX3**, EPHA4
Chr3_0519	0.60	23 (1)	0.00028	55	PAX3 (pseudogene)
Chr4_0035	2.57	132 (2)	0.00028	342	RASAL2, BRINP2
Chr4_0110	2.19	106 (2)	0.00094	338	NGFR, PHB
Chr5_0005	1.75	41 (1)	0.00049	146	CERK
Chr5_0006	2.48	292 (1)	0.00067	636	FAM19A5
Chr5_0069	4.66	224 (2)	0.00131	971	BTBD11
Chr6_0050	4.00	131 (1)	0.00094	655	MAPK10, ARHGAP24
Chr6_0079/80	0.12	12 (0)*	0.00165	69	SPOCK3
Chr6_0081	1.17	161 (17)	**0**.**00022**	480	**TLL1**

*Included as scaffolds contains annotations for neighbouring genes of TLL1 (identified by GWAS) and corresponding to a syntenic block as identified by comparison with human, mouse and opossum.

A single SNP in the region of the *NBAS* gene on chromosome 1 obtained the lowest p value from the GWAS (p = 0.00006, Table 1). This SNP along with 2 SNPs from each segregating haplotype at the PAX3 and TLL1 loci were targeted in an additional 13 control samples from WPP and one additional regressed devil from this population. The significant association at the NBAS locus disappeared following addition of further samples (Table 2). The significant associations at both TLL1 and PAX3 were retained following addition of further samples though was somewhat diminished especially for the TLL1 locus (Table 2).

**Table 2:** Results from association analysis in targeted SNPs (N = 27) as compared to results obtained from genome-wide association (N = 13).

Locus	SNP	GWAS		Targeted loci	
		Chi-square	p-value	Chi-square	p-value
NBAS	377874_T	16.34	0.00005***	0.9441	0.33120
PAX3	2290941_C	13.48	0.00024**	10.94	0.00094**
PAX3	2253019_T	16.34	0.00006***	15.48	0.00008***
TLL1	476140_C	11.8	0.00059**	5.44	0.01968*
TLL1	635881_C	11.8	0.00059**	5.347	0.02076*

*Significant at 0.05; **Significant at 0.001; ***Significant at 0.0001.

See Supplementary Material, Table S1 for a full distribution of genotypes for cases and controls.

PAX3 and TLL1 share a common physiological pathway. Both are involved in angiogenesis through regulation of intermediate targets, known as bone morphogenic proteins (BMPs). BMPs stimulate angiogenesis through activation of vascular endothelial growth factor (VEGF) in both normal development and cancer metastases^14–16^. In our regressed samples we have investigated the host genomes rather than the tumour genome, so variants identified here in PAX3 and TLL1 would be expected to play a role in the tumour microenvironment rather than the tumour itself. PAX3 expression has been implicated in a number of cancers including neuroblastoma and may enhance oncogenic potential through regulating the expression of a suite of other genes^17^.

It is possible that variants of PAX3 and TLL1 affect angiogenesis in DFTD. PAX3 is also believed to directly regulate FLT1, a VEGF receptor^18, 19^. If the process of angiogenesis is disrupted by variants of PAX3 and/or TLL1, then tumour metastasis and growth may be hindered as it has no access to the blood supply and nutrients required for growth. This may allow the infected individual more time to mount an immune response to the disease. This serves as a potential mode of action for the regression of tumours observed in devils in this study.

There may be alternative modes of action whereby PAX3 and TLL1 influence the tumour microenvironment and impact tumour progression. It remains unclear whether PAX3 and TLL1 activity is triggered by DFTD infection in regressed cases. PAX3’s involvement in a number of other cancers makes it a particularly intriguing target for further research. We cannot rule out the possibility that other candidate genes identified in the GWAS play a role in tumour regression, or that tumour lineage may be an influencing factor.

Confirmation of these results is not possible from the current dataset, however results from targeted genotyping increase confidence in the involvement of the PAX3 locus in tumour regression. Assessment of the expression of BMP and FLT (VEGF) in tissues surrounding tumours during tumour progression would enable us to understand their role in angiogenesis in DFTD. However, such samples are currently unavailable and would be very difficult to source in the field as regressing tumours are rare and sequential access to tumour and host biopsies over the time points needed for analysis is not feasible. We cannot rule out the possibility that tumour regression is not under genetic control. Taken together with recent research identifying a genetic response to DFTD infection at a population level^9^, along with identification of an immune response to DFTD in some devils in the WPP population^11^, there is mounting evidence that genetics plays a role in resistance to DFTD. The current study is preliminary in nature and we have here identified two regions worthy of further investigation and proposed a potential mode of action whereby these loci could influence tumour progression.

## Conclusion

Disruption of angiogenesis to developing tumours that slows growth and metastasis serves as a plausible model for DFTD regression in seven Tasmanian devils. Slowing of tumour growth rate may allow more time for an infected individual to mount an immune response. Tumour regression is expected to be a complex trait and many other factors and genes may be involved in this rare ability. Future work should aim to identify the frequency of this phenotype in the wild. Given the small number of animals that have recovered from the disease, it is likely to be very rare. However, the frequency may increase over time due to strong selection pressure from the disease. Confirmation of the involvement of PAX3 in DFTD regression may lead to novel treatments for this devastating disease.

## Materials and Methods

### Samples

We have sequenced the genomes of 12 devils from WPP – six of these (five females, one male) were individuals with regressed tumours that survived in the wild disease-free for some time (Table 3). Five devils (two females, three males) who had succumbed to DFTD at a young age were selected as controls. One female sample was discarded from the genome-wide association analysis as infection status was ambiguous, although genomic data from this sample was utilised for identification of variants. Devil samples for whole genome sequencing were collected in the field from WPP with approval from the University of Tasmania’s Animal Ethics Committee (A0010296 and A0013326) and from the Tasmanian Department of Primary Industries and Water (TFA15214, TFA08211, TFA14228, TFA13924). All methods were performed according to the relevant guidelines and regulations. In addition, we have utilised genome sequences from a previous study (GenBank: GCA_000219685.1, ref. 20). These two animals consist of a south-eastern male devil who succumbed to DFTD at a young age and a north-western male devil who survived a vaccination trial though eventually succumbed when re-infected in a laboratory trial.

**Table 3:** Details of 11 West Pencil Pine samples used in the genome-wide association study.

Infection status	Sex	Y.O.B.	Age 1^st^ infected	Diagnostic criteria	Comments
Regressed	F	2006	5	Visual inspection	Survived in the wild until age 5 DFTD free
Regressed	F	2006	5	Visual inspection	Survived in the wild until age 6 DFTD free
Regressed	F	2008	3	Histopathology/cytology	Tumour regressed for ~6 months
Regressed	F	2009	3	Visual inspection	Tumour regressed for ~2 years
Regressed	F	2006	3	Histopathology/cytology	Tumour regressed for ~6 months
Regressed	M	2009	3	Cytology	Survived in wild until age 7 DFTD free
DFTD-infected	M	2011	18 mths	Histopathology/cytology	Succumbed
DFTD-infected	F	2011	15 mths	Histopathology/cytology	Succumbed
DFTD-infected	M	2010	18 mths	Histopathology/cytology	Succumbed
DFTD-infected	F	2011	18 mths	Histopathology/cytology	Succumbed
DFTD-infected	F	2010	15 mths	Histopathology/cytology	Succumbed

Nb. When a tumour is detected within the mouth on sampling then no biopsy or fine needle aspiration is possible therefore visual inspection scores are used to assign infection status. As sampling took place at 3 month intervals, periods of tumour regression and survival are approximate. Y.O.B. – year of birth. Ages at first infection are given in years except for those given in months (mths).

### Sequencing and variant discovery

Tissue (ear biopsy) was obtained and DNA extracted using a phenol-chloroform extraction method^21^. The 12 Tasmanian devil genomes were sequenced by the Ramaciotti Centre for Genomics at the University of New South Wales, Kensington, with 100 base pair (bp) paired-end libraries using Truseq library preparation kits on an Illumina HiSeq 2000. An initial seven samples were sequenced in a separate lane each with another five samples sequenced across three lanes. The average read depth coverage was 10–15 times. Genome sequences were deposited in the European Nucleotide Archive (accession numbers: ERS682204-ERS682210; ERS1202857-ERS1202861). Re-sequenced genomes were aligned to the Tasmanian devil reference genome assembly version 7.0, (GenBank: GCA_000189315.1, ref. 13) using Burrows-Wheeler Aligner version 0.6.2^22^ with default parameters for paired-end data.

### SNPs and INDELs

Polymorphic sites across the entire genome were identified using SAMtools mpileup version 0.1.19^23^, including only bases and reads with minimum base and mapping quality scores of 20, respectively. Pileup was generated at all sites to allow homozygous reference genotypes to be included in the dataset if at least one sample displayed a non-reference allele after quality filtering. PCR duplicates were removed using SAMtools and local realignment around insertions and deletions was performed using GATK IndelRealigner version 2.7^24^. Insertions and deletions (INDELs) were detected using SAMtools^23^ with a coefficient of 50 based on a phred-scaled probability score used to scale down mapping quality of reads containing multiple mismatches. Variant call format files (.vcf) for individual animals were merged and filtered to retain individual locus calls with quality scores greater than 20.

### Genome-wide association

Using custom perl scripts, the SNP vcf files were filtered and re-formatted into transposed PED (tped) format to be compatible with association analysis with PLINK version 1.07^25^ and Haploview version 4.2^26^. SNPs where the root mean squared mapping quality at the site was less than 20 were excluded. For inclusion in the tped, genotypes were required to be covered by at least four high quality reads, with at least two reads or 20% of covering reads (whichever was greater) supporting the non-reference allele in order to call a heterozygous SNP. To minimize calling SNPs in duplicated loci, genotype calls were only accepted where read depth was less than 2.5 times the average mapped read depth. Variant sites were filtered for a minor allele frequency of 12% (to require an allele that is seen in at least two separate samples – i.e. 26 potential alleles from 13 samples, 3/26 = 11.5) and genotyping fail rate of <1%.

An adjusted standard genome-wide association (GWAS) in PLINK^25^ was applied to variant SNPs across the genome. The −log_10_ transformed probabilities arising from the individual locus Chi-squared tests were plotted across chromosomes using Haploview^26^ to visualise regional significance of allele frequency differences between cases (regressed devils) and controls (non-regressed devils). Due to the large number of loci in the analysis relative to the numbers of cases and controls, a second association analysis pruned the variant sites for local linkage disequilibrium (LD) using PLINK^25^. This removed SNPs in 50 bp windows where loci had LD of greater than 0.8.

### Mutation detection

Within regions of association, the available genome annotation information was combined with SNP and INDEL variants detected in our sample set. Annotated genes in devil as well as the predicted locations of genes in devil based on the human and mouse genomes were obtained from the University of California Santa Cruz Genome Browser (http://genome.ucsc.edu/; refs 27 and 28) and corresponding protein sequences were downloaded from Ensembl release 82 (www.ensembl.org; ref. 29). With these, predictions on the functional consequences of variants were made. The concordances of genomic locations of SNPs and INDELS with coding regions or untranslated regions were recorded. Next, their potential to change protein sequence (SNPs only) was assessed by translating the sequence with and without the variant in relation to the known reading frame of the protein. SNPs in conserved regions were identified from comparisons with 33 placental mammals using phastCons and phylop^30, 31^ and converted to devil coordinates using liftOver^27^.

Associated reference scaffolds arising from the GWAS were investigated further. At this time the Tasmanian devil reference genome v7.0^13^ does not have scaffolds that have been fully ordered and oriented. Instead, the reference genome comprises 35,974 scaffold sequences presumptively mapped to six autosomes and an X chromosome. We cannot conclusively identify all of the candidate genes in an associated region. To compensate for the lack of reference contiguity, significant SNPs occurring on smaller scaffolds with annotated genes were further examined by assessing synteny within more complete reference genomes including human (hg38)^32^, mouse (GRCm38)^33^ and opossum (monDom5)^34^ for potentially neighbouring genes.

### Stratification and relatedness

Relatedness among samples was assessed by calculating the pair-wise identity by descent using PLINK^25^ to identify any underlying stratification that may influence the analysis. We employed multi-dimensional scaling to visualise the two main principal components explaining the genotypic variation among our samples to test for obvious stratification between the case and control groups. A small sample size and high relatedness estimates amongst our samples impacted the power to detect a genomic signal. For this reason we chose to investigate those 100 most significant SNPs from the GWAS as this method has previously been used to successfully map a causative mutation in a genome-wide association study^35^.

### Targeted genotyping in additional samples

Primers were designed to target SNPs in the three key regions identified from the GWAS (Table S2). An additional 13 control devils from WPP who succumbed to DFTD with no apparent host response to infection were genotyped at these SNPs to identify whether the significance of the association between cases and controls was sustained for these loci (Table S3). One additional devil with confirmed regression of DFTD was also included (Table S3). Targeted SNPs were amplified by PCR using a HotStarTaq Plus DNA Polymerase kit (Qiagen) under the following conditions: 2 uL dNTPs (10 mM), 10 uL PCR buffer (10x), 2 uL forward and reverse primers (10 mM), 0.5 uL taq polymerase (5 U) per reaction using PCR cycling times of 95°C for 5 min initial denaturation, followed by 35 cycles of 94°C for 1 min, 58°C for 1 min, 72°C for 1 min and a final extension of 72°C for 10 min. Resulting PCR product was subjected to Sanger sequencing at the Australian Genome Research Facility, Westmead, NSW, Australia. Sequences were analysed using MEGA v6.06^36^ to determine individual genotypes. A second association test was conducted in PLINK on a locus by locus basis for the five targeted SNPs including both the new samples and the original 13 samples used in the GWAS.

## Electronic supplementary material

supplementary material.pdf
