# Supplementary material for: Variants in the host genome may inhibit tumour growth in devil facial tumours: evidence from genome-wide association
Source: Sci Rep. 2017 Mar 24;7:423. doi: 10.1038/s41598-017-00439-7 (PMC5428454; doi:10.1038/s41598-017-00439-7)
Supplement: Supplementary file 1 — supplementary material.pdf [file 41598_2017_439_MOESM1_ESM.pdf]

Variants in the host genome may inhibit tumour growth in devil facial tumours: evidence from genome-wide association

Belinda Wright[1], Cali E Willet[1,3], Rodrigo Hamede[2], Menna Jones[2], Katherine Belov[1], Claire M Wade[1]*

1. School of Life and Environmental Sciences, Faculty of Veterinary Science, University of Sydney, Sydney, NSW, Australia
2. School of Biological Sciences, University of Tasmania, Hobart, Tasmania, Australia
3. Sydney Informatics Core Research Facility, University of Sydney, Sydney, New South Wales, Australia

Supplementary material

Stratification and relatedness

The mean genomic relationship across all samples was 0.14 as measured by the proportion of SNPs where one allele is shared between individuals and the proportion where two alleles are shared by descent (IBD). Two samples (one in each of the control and regressed cases groups) had a genomic relationship coefficient of 0.47 which is indicative of a first order relationship. Other animals demonstrated second order or lower relationship. Despite this, the multi-dimensional scaling plot shows no stratification in clustering of cases and controls giving us confidence that there are no underlying familial relationships which may skew our analyses (Figure 1).

As 11 of our 13 samples came from a single, fairly isolated population of Tasmanian devils, we investigated relatedness and stratification amongst our samples. There was no clustering of our cases and controls (Figure S1) giving us confidence that stratified familial relationships within these two

groups did not impact results. Of the four familial relationships implied from the assessment of IBD (one 1st order, three 2nd order relationships) three were among individuals from different groups (cases and controls). This lends further confidence that genomic differences between our study groups are not purely due to familial relationships.

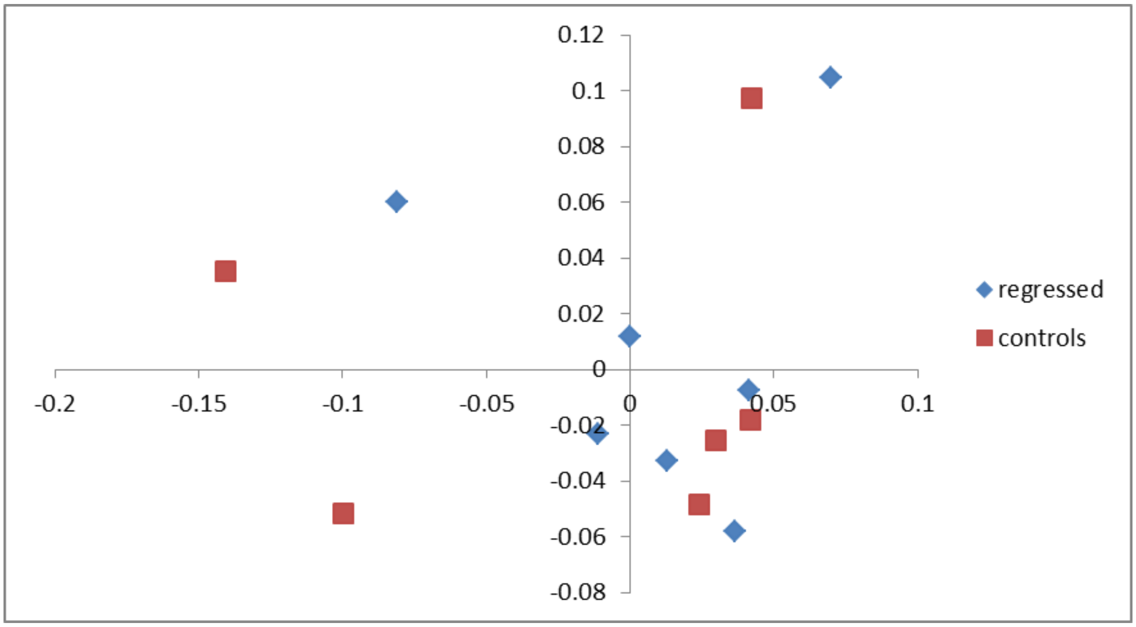

Fig S1 Multi-dimensional scaling plot of first and second principle components (C1 - X axis, C2 - Y axis) displaying no stratification for the case (regressed) and control groups.

Variant discovery

SNPs

After initial filtering on MAF of 5% there were 700,967 SNPs identified across all 14 genomes used in the SNP discovery phase. Removing one sample not suitable for the GWAS and further filtering on MAF of 12% and genotyping failure rate of 1% resulted in 466,896 SNPs. These were then pruned for LD and the resulting pruned dataset consisted of 291,263 SNPs. All reported results from the GWAS were obtained from this pruned dataset.

INDELs

There were 612190 INDEL sites identified across the genome amongst all 14 genomes compared after filtering on quality ≥ 20. INDELs within the regions of significant SNPs from the GWAS were examined for any potential impact on coding regions.

Table S1: Top 100 SNPs from genome-wide association. Minor allele frequency (MAF) and p value of test for Hardy-Weinberg equilibrium are shown for all samples and genotype (GT) ratios and observed and expected heterozygosity (H_O and H_E) are depicted for all samples as well as separated by cases and controls.

				Controls			Regressed cases			All samples			
SNP	A1	A2	MAF	GT ratio	H_O	H_E	GT ratio	H_O	H_E	GT ratio	H_O	H_E	HWE P value
Chr1_0002:319194_T	C	T	0.462	0/1/5	0.167	0.153	4/3/0	0.429	0.337	4/4/5	0.308	0.497	0.2608
Chr1_0002:343297_C	T	C	0.423	0/1/5	0.167	0.153	3/4/0	0.571	0.408	3/5/5	0.385	0.488	0.5736
Chr1_0216:2418139_T	C	T	0.269	3/1/2	0.167	0.486	0/0/7	0.000	0.000	3/1/9	0.077	0.394	0.0087
Chr1_0248:220779_T	T	G	0.462	4/2/0	0.333	0.278	0/2/5	0.286	0.245	4/4/5	0.308	0.497	0.2608
Chr1_0256:404031_T	C	T	0.269	1/5/0	0.833	0.486	0/0/7	0.000	0.000	1/5/7	0.385	0.394	1.0000
Chr1_0386:377874_T	C	T	0.423	0/0/6	0.000	0.000	5/1/1	0.143	0.337	5/1/7	0.077	0.488	0.0027
Chr2_0233:613297_A	A	G	0.385	4/1/1	0.167	0.375	0/1/6	0.143	0.133	4/2/7	0.154	0.473	0.0196
Chr2_0233:737665_G	G	C	0.385	4/1/1	0.167	0.375	0/1/6	0.143	0.133	4/2/7	0.154	0.473	0.0196
Chr2_0238:802658_T	C	T	0.269	2/3/1	0.500	0.486	0/0/7	0.000	0.000	2/3/8	0.231	0.394	0.1652
Chr2_0280:4111_A	A	C	0.385	3/3/0	0.500	0.375	0/1/6	0.143	0.133	3/4/6	0.308	0.473	0.2557
Chr2_0398:2032573_G	A	G	0.385	3/3/0	0.500	0.375	0/1/6	0.143	0.133	3/4/6	0.308	0.473	0.2557
Chr2_0398:2173106_T	C	T	0.385	3/3/0	0.500	0.375	0/1/6	0.143	0.133	3/4/6	0.308	0.473	0.2557
Chr2_0398:2204348_A	G	A	0.385	3/3/0	0.500	0.375	0/1/6	0.143	0.133	3/4/6	0.308	0.473	0.2557
Chr2_0398:2211181_C	T	C	0.385	3/3/0	0.500	0.375	0/1/6	0.143	0.133	3/4/6	0.308	0.473	0.2557
Chr2_1150:10767_A	A	C	0.385	3/3/0	0.500	0.375	0/1/6	0.143	0.133	3/4/6	0.308	0.473	0.2557
Chr2_1241:60910_A	G	A	0.346	0/0/6	0.000	0.000	2/5/0	0.714	0.459	2/5/6	0.385	0.453	0.5782
Chr2_1775:16162_A	A	G	0.346	0/0/6	0.000	0.000	4/1/2	0.143	0.459	4/1/8	0.077	0.453	0.0041
Chr2_2152:1050_T	G	T	0.269	1/5/0	0.833	0.486	0/0/7	0.000	0.000	1/5/7	0.385	0.394	1.0000
Chr3_0006:836701_T	C	T	0.346	0/0/6	0.000	0.000	3/3/1	0.429	0.459	3/3/7	0.231	0.453	0.0920
Chr3_0048:1413966_T	A	T	0.423	0/1/5	0.167	0.153	4/2/1	0.286	0.408	4/3/6	0.231	0.488	0.0855
Chr3_0321:385869_A	G	A	0.269	2/3/1	0.500	0.486	0/0/7	0.000	0.000	2/3/8	0.231	0.394	0.1652
Chr3_0369:197253_T	T	G	0.269	2/3/1	0.500	0.486	0/0/7	0.000	0.000	2/3/8	0.231	0.394	0.1652
Chr3_0369:294229_C	C	T	0.308	3/2/1	0.333	0.444	0/0/7	0.000	0.000	3/2/8	0.154	0.426	0.0334
Chr3_0388:2253019_T	T	A	0.346	4/1/1	0.167	0.375	0/0/7	0.000	0.000	4/1/8	0.077	0.453	0.0041
Chr3_0388:2275253_T	T	C	0.308	3/2/1	0.333	0.444	0/0/7	0.000	0.000	3/2/8	0.154	0.426	0.0334

Chr3_0388:2284528_A	A	G	0.346	4/1/1	0.167	0.375	0/0/7	0.000	0.000	4/1/8	0.077	0.453	0.0041
Chr3_0388:2290941_C	C	T	0.308	3/2/1	0.333	0.444	0/0/7	0.000	0.000	3/2/8	0.154	0.426	0.0334
Chr3_0388:2306297_T	T	C	0.346	4/1/1	0.167	0.375	0/0/7	0.000	0.000	4/1/8	0.077	0.453	0.0041
Chr3_0388:2311529_A	A	G	0.308	3/2/1	0.333	0.444	0/0/7	0.000	0.000	3/2/8	0.154	0.426	0.0334
Chr3_0388:2326809_C	C	T	0.346	4/1/1	0.167	0.375	0/0/7	0.000	0.000	4/1/8	0.077	0.453	0.0041
Chr3_0388:2332752_C	C	T	0.308	3/2/1	0.333	0.444	0/0/7	0.000	0.000	3/2/8	0.154	0.426	0.0334
Chr3_0388:2347209_C	C	T	0.308	3/2/1	0.333	0.444	0/0/7	0.000	0.000	3/2/8	0.154	0.426	0.0334
Chr3_0388:2379211_T	T	G	0.308	3/2/1	0.333	0.444	0/0/7	0.000	0.000	3/2/8	0.154	0.426	0.0334
Chr3_0388:2429182_G	G	A	0.308	3/2/1	0.333	0.444	0/0/7	0.000	0.000	3/2/8	0.154	0.426	0.0334
Chr3_0388:2450779_G	G	A	0.346	4/1/1	0.167	0.375	0/0/7	0.000	0.000	4/1/8	0.077	0.453	0.0041
Chr3_0388:2464777_T	T	C	0.346	4/1/1	0.167	0.375	0/0/7	0.000	0.000	4/1/8	0.077	0.453	0.0041
Chr3_0388:2475812_A	A	T	0.308	3/2/1	0.333	0.444	0/0/7	0.000	0.000	3/2/8	0.154	0.426	0.0334
Chr3_0388:2485893_G	C	G	0.269	2/3/1	0.500	0.486	0/0/7	0.000	0.000	2/3/8	0.231	0.394	0.1652
Chr3_0388:2500781_G	T	G	0.269	2/3/1	0.500	0.486	0/0/7	0.000	0.000	2/3/8	0.231	0.394	0.1652
Chr3_0388:2510258_A	G	A	0.269	2/3/1	0.500	0.486	0/0/7	0.000	0.000	2/3/8	0.231	0.394	0.1652
Chr3_0388:2544061_A	A	G	0.346	4/1/1	0.167	0.375	0/0/7	0.000	0.000	4/1/8	0.077	0.453	0.0041
Chr3_0388:2550575_C	C	A	0.308	3/2/1	0.333	0.444	0/0/7	0.000	0.000	3/2/8	0.154	0.426	0.0334
Chr3_0388:2561866_C	C	T	0.308	3/2/1	0.333	0.444	0/0/7	0.000	0.000	3/2/8	0.154	0.426	0.0334
Chr3_0388:2572580_A	A	G	0.308	3/2/1	0.333	0.444	0/0/7	0.000	0.000	3/2/8	0.154	0.426	0.0334
Chr3_0388:2583161_C	C	T	0.346	4/1/1	0.167	0.375	0/0/7	0.000	0.000	4/1/8	0.077	0.453	0.0041
Chr3_0388:2587871_T	T	C	0.346	4/1/1	0.167	0.375	0/0/7	0.000	0.000	4/1/8	0.077	0.453	0.0041
Chr3_0388:2594252_A	A	C	0.308	3/2/1	0.333	0.444	0/0/7	0.000	0.000	3/2/8	0.154	0.426	0.0334
Chr3_0388:2598794_G	G	A	0.346	4/1/1	0.167	0.375	0/0/7	0.000	0.000	4/1/8	0.077	0.453	0.0041
Chr3_0388:2604460_A	A	G	0.269	3/1/2	0.167	0.486	0/0/7	0.000	0.000	3/1/9	0.077	0.394	0.0087
Chr3_0388:2619127_T	T	C	0.346	4/1/1	0.167	0.375	0/0/7	0.000	0.000	4/1/8	0.077	0.453	0.0041
Chr3_0388:2619299_C	C	T	0.269	3/1/2	0.167	0.486	0/0/7	0.000	0.000	3/1/9	0.077	0.394	0.0087
Chr3_0388:2625468_C	C	T	0.308	3/2/1	0.333	0.444	0/0/7	0.000	0.000	3/2/8	0.154	0.426	0.0334
Chr3_0388:2638579_C	C	T	0.308	3/2/1	0.333	0.444	0/0/7	0.000	0.000	3/2/8	0.154	0.426	0.0334
Chr3_0388:2650722_A	A	G	0.308	3/2/1	0.333	0.444	0/0/7	0.000	0.000	3/2/8	0.154	0.426	0.0334
Chr3_0388:2660166_C	C	T	0.346	4/1/1	0.167	0.375	0/0/7	0.000	0.000	4/1/8	0.077	0.453	0.0041
Chr3_0388:2668393_C	C	T	0.308	3/2/1	0.333	0.444	0/0/7	0.000	0.000	3/2/8	0.154	0.426	0.0334

Chr3_0388:2733566_A	A	T	0.308	3/2/1	0.333	0.444	0/0/7	0.000	0.000	3/2/8	0.154	0.426	0.0334
Chr3_0388:3285036_G	G	A	0.269	2/3/1	0.500	0.486	0/0/7	0.000	0.000	2/3/8	0.231	0.394	0.1652
Chr3_0388:3704055_C	C	A	0.269	2/3/1	0.500	0.486	0/0/7	0.000	0.000	2/3/8	0.231	0.394	0.1652
Chr3_0388:3828025_A	A	C	0.269	2/3/1	0.500	0.486	0/0/7	0.000	0.000	2/3/8	0.231	0.394	0.1652
Chr3_0388:3846036_A	A	T	0.269	2/3/1	0.500	0.486	0/0/7	0.000	0.000	2/3/8	0.231	0.394	0.1652
Chr3_0512:706_G	G	A	0.269	2/3/1	0.500	0.486	0/0/7	0.000	0.000	2/3/8	0.231	0.394	0.1652
Chr3_0519:497777_T	T	A	0.308	3/2/1	0.333	0.444	0/0/7	0.000	0.000	3/2/8	0.154	0.426	0.0334
Chr3_2772:286_A	A	G	0.308	3/2/1	0.333	0.444	0/0/7	0.000	0.000	3/2/8	0.154	0.426	0.0334
Chr4_0035:1337774_T	T	A	0.269	2/3/1	0.500	0.486	0/0/7	0.000	0.000	2/3/8	0.231	0.394	0.1652
Chr4_0035:643556_T	T	C	0.308	3/2/1	0.333	0.444	0/0/7	0.000	0.000	3/2/8	0.154	0.426	0.0334
Chr4_0110:536495_T	T	A	0.269	3/1/2	0.167	0.486	0/0/7	0.000	0.000	3/1/9	0.077	0.394	0.0087
Chr4_0110:543210_A	A	C	0.269	3/1/2	0.167	0.486	0/0/7	0.000	0.000	3/1/9	0.077	0.394	0.0087
Chr4_0301:1026447_G	G	A	0.462	4/2/0	0.333	0.278	0/2/5	0.286	0.245	4/4/5	0.308	0.497	0.2608
Chr4_0302:675579_G	G	C	0.385	4/1/1	0.167	0.375	0/1/6	0.143	0.133	4/2/7	0.154	0.473	0.0196
Chr5_0005:437062_G	G	A	0.462	4/2/0	0.333	0.278	0/2/5	0.286	0.245	4/4/5	0.308	0.497	0.2608
Chr5_0006:2215733_C	T	C	0.346	0/0/6	0.000	0.000	3/3/1	0.429	0.459	3/3/7	0.231	0.453	0.0920
Chr5_0069:1571747_T	C	T	0.423	0/1/5	0.167	0.153	3/4/0	0.571	0.408	3/5/5	0.385	0.488	0.5736
Chr5_0069:1616335_A	G	A	0.423	0/1/5	0.167	0.153	3/4/0	0.571	0.408	3/5/5	0.385	0.488	0.5736
Chr5_0145:32969_C	T	C	0.346	0/0/6	0.000	0.000	2/5/0	0.714	0.459	2/5/6	0.385	0.453	0.5782
Chr5_2328:912_G	G	A	0.423	0/1/5	0.167	0.153	3/4/0	0.571	0.408	3/5/5	0.385	0.488	0.5736
Chr6_0050:185261_T	G	T	0.269	2/3/1	0.500	0.486	0/0/7	0.000	0.000	2/3/8	0.231	0.394	0.1652
Chr6_0081:1099040_G	G	A	0.423	0/1/5	0.167	0.153	3/4/0	0.571	0.408	3/5/5	0.385	0.488	0.5736
Chr6_0081:269543_A	A	G	0.346	0/0/6	0.000	0.000	3/3/1	0.429	0.459	3/3/7	0.231	0.453	0.0920
Chr6_0081:338939_G	G	T	0.346	0/0/6	0.000	0.000	3/3/1	0.429	0.459	3/3/7	0.231	0.453	0.0920
Chr6_0081:415652_A	A	G	0.346	0/0/6	0.000	0.000	3/3/1	0.429	0.459	3/3/7	0.231	0.453	0.0920
Chr6_0081:433138_G	G	A	0.346	0/0/6	0.000	0.000	3/3/1	0.429	0.459	3/3/7	0.231	0.453	0.0920
Chr6_0081:438380_A	A	G	0.346	0/0/6	0.000	0.000	3/3/1	0.429	0.459	3/3/7	0.231	0.453	0.0920
Chr6_0081:456505_C	C	T	0.346	0/0/6	0.000	0.000	3/3/1	0.429	0.459	3/3/7	0.231	0.453	0.0920
Chr6_0081:476140_C	C	T	0.346	0/0/6	0.000	0.000	3/3/1	0.429	0.459	3/3/7	0.231	0.453	0.0920
Chr6_0081:492347_G	G	A	0.346	0/0/6	0.000	0.000	3/3/1	0.429	0.459	3/3/7	0.231	0.453	0.0920
Chr6_0081:556198_T	T	A	0.346	0/0/6	0.000	0.000	3/3/1	0.429	0.459	3/3/7	0.231	0.453	0.0920

Chr6_0081:572557_C	C	T	0.346	0/0/6	0.000	0.000	3/3/1	0.429	0.459	3/3/7	0.231	0.453	0.0920
Chr6_0081:597647_A	A	C	0.346	0/0/6	0.000	0.000	3/3/1	0.429	0.459	3/3/7	0.231	0.453	0.0920
Chr6_0081:619210_A	A	G	0.385	0/0/6	0.000	0.000	4/2/1	0.286	0.408	4/2/7	0.154	0.473	0.0196
Chr6_0081:635881_C	C	T	0.346	0/0/6	0.000	0.000	3/3/1	0.429	0.459	3/3/7	0.231	0.453	0.0920
Chr6_0081:668083_G	G	A	0.385	0/0/6	0.000	0.000	4/2/1	0.286	0.408	4/2/7	0.154	0.473	0.0196
Chr6_0081:850942_T	T	C	0.462	0/1/5	0.167	0.153	4/3/0	0.429	0.337	4/4/5	0.308	0.497	0.2608
Chr6_0081:877431_A	A	G	0.423	0/1/5	0.167	0.153	3/4/0	0.571	0.408	3/5/5	0.385	0.488	0.5736
Chr6_0099:1377218_G	G	C	0.462	5/0/1	0.000	0.278	0/2/5	0.286	0.245	5/2/6	0.154	0.497	0.0206
Chr6_0328:42978_C	C	G	0.346	0/0/6	0.000	0.000	3/3/1	0.429	0.459	3/3/7	0.231	0.453	0.0920
Chr6_0597:47260_T	T	C	0.346	0/0/6	0.000	0.000	3/3/1	0.429	0.459	3/3/7	0.231	0.453	0.0920
Chr6_0630:19576_G	G	A	0.346	0/0/6	0.000	0.000	3/3/1	0.429	0.459	3/3/7	0.231	0.453	0.0920
Chr6_0749:34101_C	C	A	0.346	0/0/6	0.000	0.000	3/3/1	0.429	0.459	3/3/7	0.231	0.453	0.0920
Chr6_1014:830_G	G	T	0.346	0/0/6	0.000	0.000	3/3/1	0.429	0.459	3/3/7	0.231	0.453	0.0920

Table S2 Primer details for additional targeted markers.

Chr	SNP	Primer	sequence (5'-3')	Tm^{o}C	Ensembl location
1	377874_T	NBAS_fwd	CTCCCTCAGATCCCCTTTCT	59.6	GL834798:377759-377778
		NBAS_rev	GAATGCTGCAGAAGATGTGC	59.6	GL834798:377984-378003
3	2253019_T	PAX3.1_fwd	TGCCTGAGAACACGTTTTGAT	60.7	GL849910:2252793-2252813
		PAX3.1_rev	CCAGTGGGATTAGAGCCTTTT	59.6	GL849910:2253165-2253185
3	2290941_C	PAX3.2_fwd	CTGGCATCTGCTCTTTGATCT	59.6	GL849910:2290670-2290690
		PAX3.2_rev	TGGTAAGTCTGGCTCCGAAT	59.7	GL849910:2291024-2291043
6	476140_C	TLL1.1_fwd	TTCACAACGGACTTGGACAG	59.7	GL864805:475983-476002
		TLL1.1_rev	AGATGTAGACCCAGACATGATACC	58.4	GL864805:476196-476219
6	635881_C	TLL1.2_fwd	AATGGTGGGATGCTGAAAAT	59.2	GL864805:635757-635776
		TLL1.2_rev	GGATAAGAAGATTTGGCTCTGG	59.2	GL864805:635978-635999

Table S3 Additional sample details used in targeted genotyping analysis.

Infection status	Sex	Y.O.B	Age 1st infected	Diagnostic criteria
Regressed	F	2006	5	Histopathology/cytology
DFTD-infected	F	2006	5	Histopathology/cytology
DFTD-infected	M	2005	2	Visual inspection
DFTD-infected	M	2006	2	Visual inspection
DFTD-infected	M	2003	5	Histopathology
DFTD-infected	F	2007	2	Visual inspection
DFTD-infected	M	2006	3	Cytology
DFTD-infected	F	2007	4	Visual inspection
DFTD-infected	M	2006	2	Histopathology
DFTD-infected	F	2006	3	Visual inspection
DFTD-infected	F	2006	2	Visual inspection
DFTD-infected	M	2006	3	Visual inspection
DFTD-infected	F	2008	2	Histopathology/cytology
DFTD-infected	F	2006	4	Histopathology/cytology
